# 4-Hydroxychalcone Attenuates Hyperaldosteronism, Inflammation, and Renal Injury in Cryptochrome-Null Mice

**DOI:** 10.1155/2014/603415

**Published:** 2014-06-09

**Authors:** Qi Qu, Bingguang Dai, Bo Yang, Xuelian Li, Yimin Liu, Fuling Zhang

**Affiliations:** ^1^Department of Cardiovascular Surgery, The Fourth People's Hospital of Jinan City, Jinan 250031, China; ^2^Department of Cardiovascular Surgery, Qilu Children's Hospital of Shandong University, Jinan 250031, China

## Abstract

In the present study, we aimed to investigate the preventive effects of 4-hydroxychalcone (4HCH) on resistant hypertension. We used cryptochrome-null mice, which characteristically show high plasma aldosterone levels, inflammation, and renal injury. The cryptochrome-null mice received high-salt treatment and were treated orally with 4HCH 10 mg/kg, 4HCH 20 mg/kg, and 4HCH 40 mg/kg, respectively. The salt administration in cryptochrome-null mice is able to induce an increase in systolic pressure which is associated with hyperaldosteronism, inflammation, and kidney injury. Treatment with 40 mg/kg 4HCH reduced systolic hypertension, serum IL-1**β**, and TNF-**α** levels and suppressed the activation of nuclear factor kappa-light-chain-enhancer of activated B cells (NF-**κ**B) and renal injury. The impact of 4HCH on the hyperaldosteronism, inflammation, and kidney injury provides new insights for future development of therapeutic strategies in resistant hypertension.

## 1. Introduction

Resistant hypertension (RH) refers broadly to high blood pressure that is resistant to pharmacologic therapy. Although the etiology of resistant hypertension is almost always multifactorial, aldosterone excess has been shown to contribute importantly to the development of RH [1]. There is evidence that aldosterone can promote endothelial dysfunction and induce vascular inflammation, vascular and myocardial fibrosis, and myocardial ischemia [2–4]. Consistent with the high degree of aldosterone excess demonstrable in patients with resistant hypertension, blockade of aldosterone has been demonstrated to provide particular benefit to RH [5].

Chalcones (1,3-diaryl-2-propen-1-ones) belong to the largest class of plant secondary metabolites and are considered to be precursors of flavonoids and isoflavonoids serving in plant defense mechanisms to counteract reactive oxygen species in order to survive and prevent molecular damage and damage by microorganisms, insects, and animals [6]. Chalcones and their derivatives exerted a lot of biological properties [7, 8], but few previous reports referred to the ability of these classes of compounds to lower blood pressure via the blockade of aldosterone. 4-Hydroxychalcone (4HCH) is an alpha, beta-unsaturated ketone with the core structure of chalcone and one hydroxyl substituent on the 4 positions of the A ring. We present for the first time the evidence that 4HCH inhibits RH by attenuating hyperaldosteronism, inflammation, and renal injury in cryptochrome-null mice (CNM).

Cryptochrome-null mice show increased mRNA expression and protein levels of 3*β* hydroxysteroid dehydrogenase [9]. The enzyme is expressed particularly in the zona glomerulosa, where aldosterone production is known to exclusively take place. In the present study, we used cryptochrome-null mice, which characteristically show high plasma aldosterone levels, to evaluate the efficacy of 4HCH to lower blood pressure and prevent progressive hyperaldosteronism, inflammation, and renal injury.

## 2. Materials and Methods

### 2.1. Drugs

4HCH with 98% purification was obtained following the extraction and separation using a column chromatographic method [10].

### 2.2. Animals

This study was performed in accordance with the Guide for the Care and Use of Laboratory Animals. Care was taken to minimize discomfort, distress, and pain to the animals. The CNM were developed by the way introduced by Vitaterna et al. [11]. CNM received high-salt treatment. The normal salt chow had a 0.2% NaCl content, whereas the high-salt chow had a 3.15% NaCl content, and the drinking water contained 1% NaCl and 0.2% KCl.

### 2.3. Experimental Design

Forty-eight of these mice were allocated equally into 4 groups: CNM group, CNM and 4HCH-10 group, CNM and 4HCH-20 group, and CNM and 4HCH-40 group. The other 12 wild-type (WT) littermates mice were used as the control group receiving normal salt treatment. From then on, the 5 groups of mice were orally administered saline, 10 mg/kg 4HCH, 20 mg/kg 4HCH, 40 mg/kg 4HCH, and saline, respectively. 4HCH was dissolved in distilled water and administrated orally twice daily using a feeding needle for 35 days, and control group received double distilled water instead of 4HCH.

### 2.4. Blood Pressure Measurement

Blood pressure was determined in conscious, trained mice using a noninvasive computer-automated tail-cuff system (BP-98A, Softron, Shanghai, China). The average value of 12 measurements was used for data analysis.

### 2.5. Blood Biochemical Analysis

After 32 weeks of treatment, blood was obtained for biochemical analysis by cardiac puncture before the mice were killed. Serum IL-1*β*, TNF-*α*, and aldosterone were measured using a commercially available enzyme immunoassay kit (Shanghai Jinma Biological Technology, Inc., China) according to the protocol described by the manufacturer.

### 2.6. Quantification of NF-*κ*B Activity

At the end of the 32-week experimental period, kidney tissue samples were collected. Activated NF-*κ*B was quantified in kidney tissue extracts via ELISA technique using the PathScan Phospho-NF*κ*B p65 (Ser536) Sandwich ELISA Antibody Pair (Shanghai Yubo Biological Technology, Inc., China), following the manufacture's instruction. The protein expression levels of NF-*κ*B were measured by Western blot analysis.

### 2.7. Histological Examination of Kidney Sections

Kidney samples were fixed in 4% buffered formalin (pH 7.2), processed, and embedded in paraffin wax. Sections of 5 mm thickness were then generated and stained with Periodic acid–Schiff reagent for subsequent light microscope examination. Histological evaluation was performed in a blinded manner. A minimum of two slides per rat were read.

### 2.8. Statistical Analysis

The data were expressed as mean ± SEM and results were analyzed by ANOVA followed by Dunnett's *t* test. *P* < 0.05 was considered significant.

## 3. Results

### 3.1. The Effect of 4HCH on Blood Pressure of Mice

**Figure 1:**
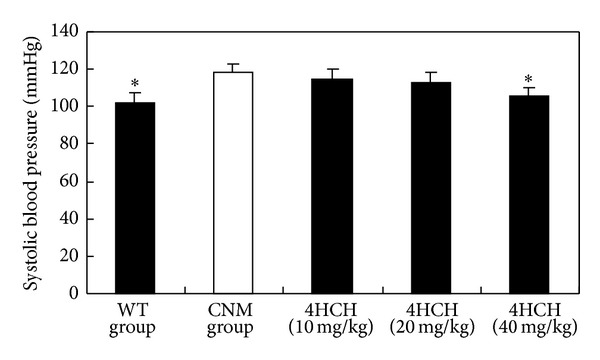
Effect of 4HCH on systolic blood pressure. Data are presented as the SEM. *P < 0.05 as compared with CNM group.

CNM have elevated blood pressure compared with their WT littermate controls (Figure 1). WT mice (*n* = 12) exhibited a systolic pressure of 103.1 ± 5.5. In contrast, CNM (*n* = 10) were hypertensive (118.0 ± 8.6). At 10 and 20 mg/kg, 4HCH did not produce a significant change from blood pressure baseline. At the higher dosage of 40 mg/kg, 4HCH lowered blood pressure in CNM (*P* < 0.05) (Figure 1).

### 3.2. The Effect of 4HCH on Serum Aldosterone Levels

**Figure 2:**
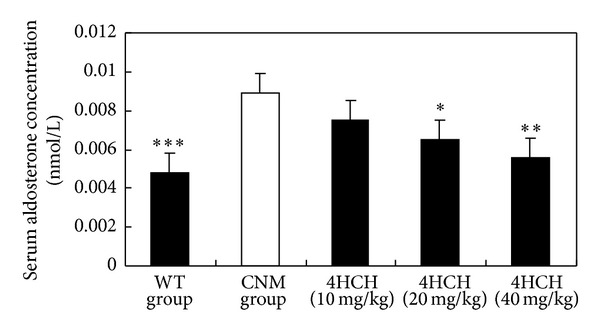
Effect of 4HCH on serum aldosterone levels. Data are presented as the SEM. *P < 0.05 as compared with CNM group, **P < 0.01 as compared with CNM group, and ***P < 0.001 as compared with CNM group.

Figure 2 shows plasma aldosterone concentrations in CNM and WT mice, respectively. Serum aldosterone levels were significantly greater in CNM compared with WT mice (*P* < 0.001). However, aldosterone levels in the 40 mg/kg 4HCH and 20 mg/kg 4HCH groups were significantly lower than those in the CNM group (*P* < 0.01 and *P* < 0.05, resp.).

### 3.3. The Effect of 4HCH on Serum IL-1*β* and TNF-*α* Level

**Figure 3:**
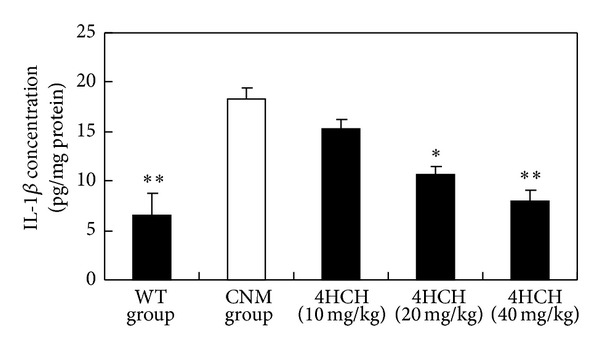
Effect of 4HCH on IL-1*β* level. Values represent the mean ± SEM. *P < 0.05 versus CNM group. **P < 0.01 versus CNM group.

**Figure 4:**
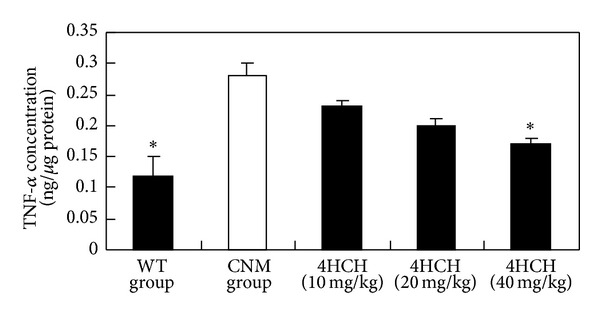
Effect of 4HCH on TNF-*α* level. Values represent the mean ± SEM. *P < 0.05 versus CNM group.

In comparison to CNM group (Figure 3), treatment with 10 and 20 mg/kg 4HCH resulted in a marked decrease in IL-1*β* levels compared with those in CNM group (*P* < 0.05 and *P* < 0.01, resp.). In addition, the levels of TNF-*α* were significantly increased in CNM group (Figure 4). 4HCH (40 mg/kg) suppressed CNM-induced TNF-*α* production (*P* < 0.05).

### 3.4. The Effect of 4HCH on Protein Expression of NF-*κ*B

**Figure 5:**
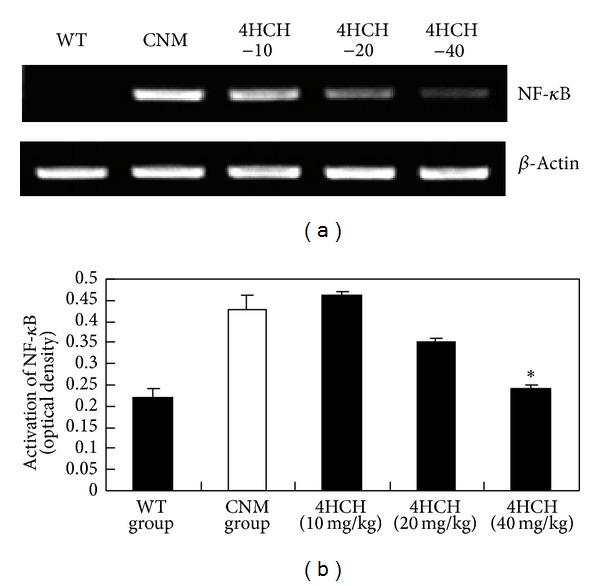
Effect of 4HCH on protein expression of NF-*κ*B. Values represent the mean ± SEM. *P < 0.05 versus CNM group.

The protein expression of NF-*κ*B represents NF-*κ*B activation. As shown in Figure 5(a), protein expression of NF-*κ*B was significantly increased in the CNM group, suggesting CNM induced a predominant increase in nuclear translocation of NF-*κ*B substantially. Conversely, level of NF-*κ*B protein decreased in the nucleus of kidney cells of 4HCH (40 mg/kg) group (Figure 5(b)).

### 3.5. The Effect of 4HCH on Renal Injury

**Figure 6:**
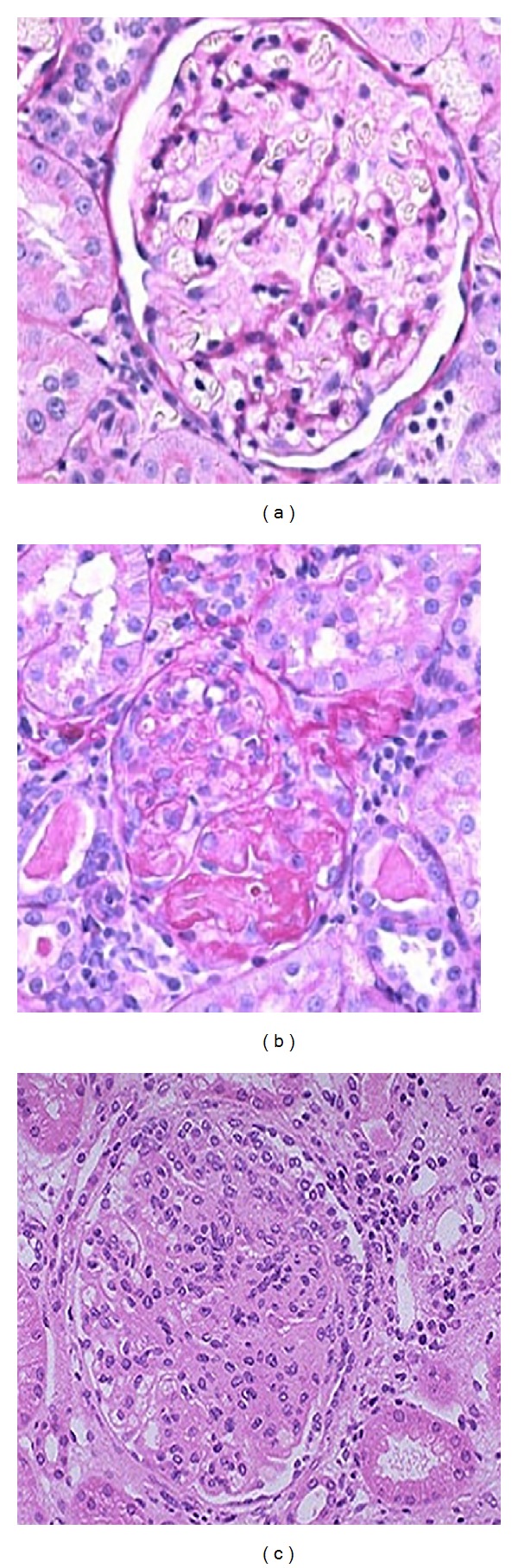
Effects of 4HCH on salt-induced kidney damage were evaluated by morphological analysis of (a) WT mice, (b) CNM, and (c) 4HCH-40 mice. Magnification ×200.

On histologic analysis, CNM with high-salt treatment developed severe glomerulosclerosis and tubulointerstitial injury and moderate tubulointerstitial infiltration with inflammatory cells (Figure 6(b)). It was dramatically reduced and became more normal in 4HCH (40 mg/kg) treated mice (Figure 6(c)).  Figure 6(a) showed that there were no marked renal abnormalities in WT mice.

## 4. Discussion

In the present study, we demonstrated that 4HCH reduces hypertension in CNM mice. This antihypertensive property of 4HCH may be explained by the attenuation of hyperaldosteronism and anti-inflammatory activities and recovery of renal injury.

A now very sizable body of literature implicates aldosterone as an important mediator of incident hypertension and severity of hypertension, and, in particular, a common cause of resistance to antihypertensive treatment [12–14]. CNM show an adrenal disorder characterized by chronic overproduction of aldosterone that persists even in the reduced plasma renin activity [9]. The levels of aldosterone measured in the current study relate well to previously published studies [11]. The studies suggest that hypertension in high-salt-treated CNM was caused by mineralocorticoid receptor activation owing to both hyperaldosteronism and high-salt exposure. Here we have clearly demonstrated 4HCH lower level of aldosterone and blood pressure. It indicated that blockade of the aldosterone pathway by 4HCH prevented the increase in systolic pressure (Figure 1).

The importance of inflammation as being central to the development of atherosclerosis has been a fundamental tenet of cardiovascular medicine [15–17]. The low-grade inflammation occurs in the vasculature in various conditions that predispose to cardiovascular disease, including hypertension [18]. We noted that the CNM in the present study developed inflammation following high-salt treatment and in the absence of elevated blood pressure. It is consistent with previous findings that renal proinflammatory response plays an important role in mediating hypertension [19–22]. In our study, we show in CNM that salt-sensitive hypertension increased serum IL-1*β* and TNF-*α* level, which are normalized by 4HCH treatment.

NF-*κ*B is a key transcription factor in the activation of genes related to proinflammatory response. It is one of the important renal mechanisms linking proinflammatory response to SS hypertension [23]. The present study demonstrates that a high-salt-induced renal activation of NF-*κ*B correlates with a significant upregulation of IL-1*β* and TNF-*α* level in CNM. Our results indicate that 32 wk of treatment with 4HCH suppressed high-salt-induced renal activation of NF-*κ*B (Figure 6). Such a mechanism contributes probably to the beneficial effect of 4HCH on hypertension in CNM.

It is known that salt-sensitive hypertension in human and experimental animal model has been associated with progressive kidney damage leading to end-stage renal disease caused by elevated inflammation [24, 25]. Consequently, in the present study, we hypothesized that the 4HCH ameliorates hypertension and the associated kidney injury. The renal protection is evident from histopathologic observations including markedly reduced tubular cast formation and fibrosis in the kidney (Figure 6). This is the first study to demonstrate that 4HCH has a direct effect on protection against salt-induced kidney injury dependent on its ability to lower blood pressure.

In conclusion, this study demonstrates that salt administration in CNM is able to induce an increase in systolic pressure which is associated with hyperaldosteronism, inflammation, and kidney injury. These arterial modifications could represent an early step in the development of resistant hypertension. These changes were all reversed by orally administrated 4HCH. The impact of 4HCH on the hyperaldosteronism, inflammation, and kidney injury provides new insights for future development of therapeutic strategies in resistant hypertension.
